# The relationship between social media use, anxiety and burden caused by coronavirus (COVID-19) in Spain

**DOI:** 10.1007/s12144-021-01802-8

**Published:** 2021-05-22

**Authors:** Julia Brailovskaia, Marta Miragall, Jürgen Margraf, Rocío Herrero, Rosa M. Baños

**Affiliations:** 1 Mental Health Research and Treatment Center, Department of Clinical Psychology and Psychotherapy, Ruhr-Universität Bochum, Massenbergstr. 9-13, 44787 Bochum, Germany; 2 Department of Personality, Evaluation, and Psychological Treatments, University of Valencia, Valencia, Spain; 3 CIBER Fisiopatología Obesidad y Nutrición (CIBEROBN), Instituto Carlos III, Madrid, Spain; 4 Instituto Polibienestar, University of Valencia, Av. Blasco Ibáñez 21, Valencia, Spain

**Keywords:** COVID-19, Social media as information source, Anxiety, Burden

## Abstract

The outbreak of COVID-19 and national restrictions to slow down its spread have significantly changed people’s everyday lives. Many people engage in intensive social media use (SMU) to stay up-to-date about the pandemic. The present study investigated the extent of SMU as source of COVID-19 information, and its relationship with anxiety and the experienced burden caused by the pandemic in Spain. Of the 221 participants, 52.5% reported to frequently use SM as information source. The use of other information sources such as print and online newspaper reports, television reports, and official governmental online sites was not associated with anxiety and burden caused by the current COVID-19 situation. However, SMU was significantly positively linked to both variables. Moreover, anxiety significantly mediated the relationship between SMU and the experienced burden. The findings show the potential negative effect of SMU on individual emotional state and behavior during the pandemic. They emphasize the significance of an accurate and conscious use of SM specifically during extraordinary circumstances such as the COVID-19 outbreak.

## Introduction

The outbreak of the coronavirus disease in December 2019 (COVID-19; severe acute respiratory syndrome coronavirus 2, SARS-CoV-2) and its rapid spread across the globe have significantly changed people’s everyday lives. Spain was one of the most severe COVID-19 affected European countries at the beginning of the pandemic (World Health Organization, 2020).

On March 14, 2020, when the national lockdown became effective in Spain (Zafra et al., 2020), a total of 6391 COVID-19 cases and 195 deaths were confirmed in the Spanish population (Hasell et al., 2020). To reduce physical contact, public institutions, non-essential shops, businesses and recreation areas were temporarily closed. All mass events were canceled and a “stay-at-home” was ordered for all residents. Leaving home was allowed only in urgent cases, such as to purchase food and medicine, to walk the dog, and for essential work (i.e., those related to the maintenance of basic social functions and/or the efficient working of the institution of state and public administrations). As far as possible, employees were advised to work remotely (The Guardian, 2020). On May 2, 2020, when the confinement measures were eased and several new rules were introduced by the Spanish government in the Official State Bulletin (Order SND/380/2020) (Ministerio de Sanidad, 2020a), a total of 216,582 COVID-19 cases and 25,100 deaths were confirmed in Spain (Hasell et al., 2020). The easing of the measures included the permission of short walks at certain hours of the day (i.e., maximum one hour per day, maximum 1 km from home, and only two persons at a time from the same household) and individual sports (i.e., in one’s home city or village).

The COVID-19 crisis causes many restrictions on daily life and is a high burden for many people. They experience uncertainty, frustration, and hopelessness that can negatively impact mental and physical health as well as decrease the willingness to adhere to the governmental rules and measures such as wearing face masks and maintaining of “social distance” (Galea et al., 2020). Considering these negative consequences and the importance of adherence to the rules to fight the pandemic, it is urgent to investigate factors that can predict the level of burden experienced by the current COVID-19 situation. This knowledge can be used to identify persons at risk for high burden, to reduce burden and to enhance resilience among the population.

Since the beginning of the pandemic, the need for offline “social distancing” contributed to an enhanced use of online social media (SM) such as Facebook, Instagram and Twitter (Gao et al., 2020). Many people engage in intensive social media use (SMU) to gain information about the current COVID-19 situation (Allington et al., 2020). While other sources of information such as print and online newspaper reports, television reports, and official governmental online sites allow only a passive consumption of filtered information, users of SM can actively participate in the creation and sharing of the content (Boyd & Ellison, 2007). Previous research described the search for information and the wish for social interaction to be among the main motives for excessive SMU (Brailovskaia et al., 2020). Both motives were shown to grow stronger after the governmental measures to slow down the COVID-19 spread were introduced (Allington et al., 2020; Gao et al., 2020).

On SM, users typically engage in intensive interactions with friends and family, but also with strangers to be up-to-date about current events in different areas. By immersing into the online world, they can at least temporarily escape daily problems and loneliness (Brailovskaia et al., 2019; Verduyn et al., 2017). The feelings of isolation due to the COVID-19 measures can be reduced by the online communication (Cellini et al., 2020; Gao et al., 2020).

Although the positive effect of online social support and the feeling of belonging when using SM are important in times of “social distancing”, earlier research reported a positive relationship between the use of social platforms and symptoms of anxiety (Atroszko et al., 2018; Brailovskaia & Margraf, 2020a; Twenge et al., 2018). The exposure to extensive unfiltered (mis)information typically provided on SM was described as one of the reasons for this finding by studies on earlier epidemics, terrorist attacks, and other extraordinary societal situations (Garfin et al., 2015; Homan et al., 2014; Thompson et al., 2017), as well as by recent research on the current COVID-19 situation (Gao et al., 2020). Even though SM providers try to control the content shared on their platforms, fake news that exaggerate and/or falsify official information can rapidly spread on SM via re-tweets on Twitter, and liking and re-sharing of content on Facebook (Budhwani & Sun, 2020; Garfin et al., 2020; Ng et al., 2018; Pennycook et al., 2020). The consumption of the unfiltered (mis)information and consequential interaction with other users can contribute to an emotional contagion and to a rapid spread of anxiety (Garfin et al., 2015; Wang et al., 2019). This can have negative consequences for the individual and the society. Notably, recent research described that anxiety positively predicts the level of burden experienced by COVID-19 (Brailovskaia & Margraf, 2020b).

Against the presented background, the specific COVID-19 situation in Spain at the beginning of May – when the extensive new governmental measures should be well-known by all citizens in order to fight against the pandemic and not being penalized by the police –, and the importance of understanding predictors of burden by the current COVID-19 situation, the present study aimed (1) to assess the extent of SMU as COVID-19 information source in comparison to other sources such as newspaper articles (offline and online), news reports on television and official governmental online sites in Spain. And further (2) to investigate the following hypotheses: It is assumed that SMU as COVID-19 information source is positively related to anxiety (Hypothesis 1a) and the experience of burden (Hypothesis 1b). Moreover, anxiety is expected to serve as a mediator of the relationship between SMU and burden caused by COVID-19 (Hypothesis 2).

## Methods

### Procedure and Participants

The overall sample is comprised of 221 participants from Spain. Table 1 presents the socio-demographic characteristics of the sample. Power analyses using the G*Power program, version 3.1 (Faul et al., 2009) indicated that the sample size is sufficient for valid results (power > .80, *α* = .05, effect size: *f*^*2*^ = .15; cf., Mayr et al., 2007).

**Table 1:** Socio-demographic characteristics of the investigated sample

	% (*n*)	*M (SD)*	*Min–Max*
Age		36.11 (13.57)	18–68
*Gender*			
Men	17.6 (39)		
Women	82.4 (182)		
*Occupation*			
Employed	68.3 (151)		
Student	24 (53)		
Unemployed	7.7 (17)		
*Marital status*			
Single	38 (84)		
In a romantic relationship	37.1 (82)		
Married	24.9 (55)		
*COVID-19*			
Participant has been tested positive	1.4 (3)		
Family members or friends have been tested positive	16.3 (36)		

Notes. *N* = 221; M = Mean, SD=Standard Deviation, Min = Minimum, Max = Maximum; COVID-19 = coronavirus disease 2019

The link of the online survey in Spanish language was sent to overall 493 persons on the May 3, 2020 through the Qualtrics platform (https://www.qualtrics.com). They were recruited through invitation flyers uploaded on different social platforms (i.e., Facebook, Twitter, Instagram) at the beginning of the pandemic in Spain and agreed to participate in online surveys of the University of Valencia (Spain). The survey was completed between May 3 and May 6, 2020 by 221 out of the 493 (44.8%) participants who received the link; ten gift cards worth 40 Euros each were offered through a raffle to encourage the participation. Inclusion criteria were: (1) age ≥ 18 years and (2) be located in Spain at the time of confinement; no exclusion criteria were established. Average completion time was 25–30 min. All participants were provided instruction and gave informed consent to participate via an online form. The study was approved by the Ethical Committee of the University of Valencia (register number: 1593681212393) and conducted in accordance with the principles outlined in the Declaration of Helsinki (World Medical Association, 2013).

### Measures

#### Socio-Demographic Characteristics

Gender, age, marital status, occupational situation, the percentage of individuals affected by COVID-19 in terms of health were assessed.

#### Anxiety Symptomatology

The Generalized Anxiety Disorder Questionnaire-2 (GAD-2; Spanish version: García-Campayo et al., 2012; original version: Kroenke et al., 2007) was used to assess anxiety symptomatology. It includes two items that are rated on a 4-point Likert-type scale (0 = *not at all*, 3 = *nearly every day*; scale reliability: Cronbach’s *α* = .781). The total score ranges from 0 to 6. A score of ≥ 3 on the GAD-2 has been identified as a cut-off for identifying clinically significant anxiety symptomatology.

#### COVID-19 Specific Media Use

Participants rated the frequency of their use of various forms of media to inform themselves about the current COVID-19 situation: 1) news reports on television, 2) newspaper articles (print media), 3) online newspaper articles, 4) official sites of the federal government and authorities, and 5) social media (e.g., Facebook, Twitter) on a 7-point Likert-type scale (1 = *not at all*, 7 = *intensively*). The items were formulated by the principal investigators in the Spanish language.

#### Burden Caused by COVID-19

The experience of burden caused by COVID-19 was measured by overall eight items. The COVID-19 Burden Scale (original version: Brailovskaia & Margraf, 2020b) that includes six items (e.g., “I am burdened by the current social situation”, “I feel restricted in my everyday life”, “I feel socially isolated”) was translated by the customary translation-backtranslation-modification procedure into the Spanish language (Berry, 1989). In addition, the principal investigators added two further items (original language: Spanish) to the available six items (i.e., “My present situation has some advantages”, “The current social situation has improved my social interaction with the people I live with”). All items are rated on a 7-point Likert-type scale (1 = *I do not agree*, 7 = *I totally agree*; scale reliability: *α* = .685). The higher the sum score, the higher the level of burden.

### Statistical Analyses

Statistical analyses were conducted using SPSS 24 and the macro Process version 2.16.1 (www.processmacro.org/index.html; Hayes, 2013). After descriptive analyses, dependent t-tests were calculated to compare the extent of SMU with the extent of use of news reports on television, newspaper articles (print media), online newspaper articles, and official sites of the federal government and authorities as source of COVID-19 information. Cohen’s d was included as effect size measure. Zero-order bivariate correlation analyses assessed the relationship between the information sources, anxiety and burden caused by COVID-19. Based on the findings of the correlation analyses a mediation model was calculated (Process: model 4). The model included SMU as source of COVID-19 information as predictor, anxiety as mediator, and burden caused by COVID-19 as outcome. Path *a* denoted the association between SMU and anxiety; the link between anxiety and burden was denoted by path *b*. The combined effect of path *a* and path *b* represented the indirect effect (*ab*). Path *c* (the total effect) denoted the basic relationship between SMU and burden, while the association between SMU and burden after the inclusion of anxiety in the model was denoted by path *c’* (the direct effect). The bootstrapping procedure (10,000 samples) that provides accelerated confidence intervals (*CI* 95%) assessed the mediation effect.

## Results

Table 2 presents the descriptive statistics of the investigated variables.

**Table 2:** Descriptive statistics and correlations of the investigated variables

		*Descriptive Statistics*				*Correlations*	
		Rating					
		“1″	“2” and “3″	“4” and “5″	“6” and “7″		
	*M (SD)*	% (*n*)	% (*n*)	% (*n*)	% (*n*)	(1)	(2)
(1) Burden by COVID-19	29.01 (7.84)						.375**
(2) Anxiety	1.98 (1.60)						
*COVID-19 specific media use*							
(3) Television	4.00 (2.06)	15.40 (34)	28.10 (62)	26.70 (59)	29.90 (66)	−.032	.028
(4) Newspaper (print media)	1.74 (1.48)	71.90 (159)	16.30 (36)	6.30 (14)	5.40 (12)	.036	.007
(5) Newspaper (online media)	4.25 (2.04)	14.00 (31)	21.70 (48)	32.60 (72)	31.70 (70)	.098	.099
(6) Official sites	3.84 (2.12)	18.60 (41)	29.00 (64)	25.80 (57)	26.70 (59)	−.011	.075
(7) Social Media	3.66 (2.09)	21.30 (47)	26.20 (58)	28.50 (63)	24.00 (53)	.171*	.229**

*Notes. N* = 221; “1” = *not at all*, “7” = *intensively*; COVID-19 = coronavirus disease 2019; **p < .01, *p < .05

Most participants frequently used online newspaper reports as source of COVID-19 information (range “4” to “7”: 64.2%), followed by news reports on television (range “4” to “7”: 56.6%). About the half of the participants used frequently SM and official federal sites (both: range “4” to “7”: 52.5%). And 11.8% frequently used newspaper articles (print media) (range “4” to “7) as information source about COVID-19. More specifically, considering the mean usage frequency, t-tests revealed that participants tended to use SM more frequently than newspaper reports in print media as source of COVID-19 information, *t*(220) =11.215, *p* < .001, d = 1.062, and less frequently than online newspaper articles, *t*(220) = −3.993, *p* < .001, d = 0.287. No significant differences were found between the use of SM and of television reports, *t*(220) = −1.696, *p* = .091, d = 0.161, as well as of official federal sites, *t*(220) = −.996, *p* = .320, d = 0.084.

As shown in Table 2, burden caused by COVID-19 was significantly positively correlated with anxiety. Furthermore, SM was the only source of COVID-19 information that was significantly positively correlated with burden and anxiety (see Table 2) (confirmation of Hypothesis 1a and of Hypothesis 1b).

**Fig. 1:**
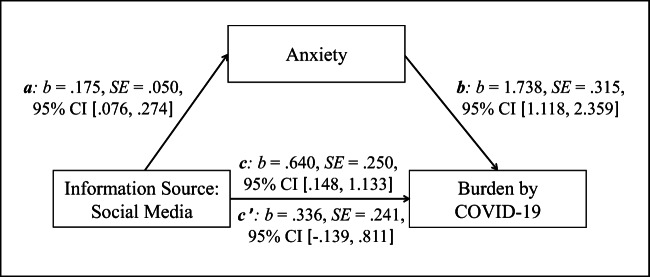
Mediation model with social media use as source of COVID-19 information (predictor), anxiety (mediator), and burden caused by COVID-19 (outcome). *Notes. N = 221; a* = path of predictor to mediator; *b* = path of mediator to outcome; *c* = path of predictor to outcome, without inclusion of mediator (total effect); *c’* = path of predictor to outcome including mediator (direct effect)

Based on this finding, only one bootstrapped mediation analysis was calculated. As shown in Fig. 1, anxiety significantly mediated the relationship between SMU and burden (confirmation of Hypothesis 2). The basic relationship between SMU (predictor) and burden (outcome) was significant (total effect, *c: p* = .011). The association between SMU and anxiety (mediator) (*a*: *p* < .001), as well as the relationship between anxiety and burden (*b*: *p* < .001) were also significant. After the inclusion of anxiety in the model, the relationship between SMU and burden was not significant (direct effect, *c’: p* = .164). The indirect effect was significant (*ab: b* = .304, SE = .117, 95% CI [.106, .557]).

## Discussion

Previous research from Europe (Brailovskaia & Margraf, 2020a) and from the U.S. (Twenge et al., 2018) reported an increase of social media use in the past decade that is accompanied by a decrease of well-being. The present study from Spain confirms and extents the available findings on the potential negative effect of SMU during the pandemic.

The outbreak of COVID-19 changed people’s everyday lifes around the globe. Due to the need for “social distancing”, a large part of daily interaction with family, friends and colleagues was transferred into the online world. As a consequence of the increased leisure time due to home-schooling and work loss, many people, especially adolescents and young adults, enhanced the use of SM such as Facebook, Instagram and Twitter (Cellini et al., 2020; Gao et al., 2020).

The current results stem from a Spanish sample that was assessed between May 3 and May 6, 2020, when the confinement measures were eased and several new governmental measures regarding the stay-home-order were announced by the Spanish government. The findings reveal that in order to stay up-to-date about the COVID-19 situation, SM were used as frequently as official federal government sites and television reports and more frequently than newspaper reports in print media. Only online newspaper articles seem to be used more frequently than SM as a COVID-19 information source. However, in contrast to all the other information sources, SM allow users to actively participate in the creation, modification and sharing of information and to interact about the content with other users. As a consequence, a rapid spread of misinformation and fake news cannot be excluded (Allington et al., 2020).

As revealed by the present findings, this can negatively impact users’ well-being. When looking at the sources of information, only the use of SM as a COVID-19 information source was positively associated with anxiety (confirmation of Hypothesis 1a) and with the experience of burden (confirmation of Hypothesis 1b). None of the other investigated information sources (i.e., online and offline newspaper reports, television reports, official sites) was significantly linked to both variables. Typically, the experience of anxiety in uncertain situations negatively influences the individual resilience and coping processes. It contributes to frustration, feelings of helplessness and hopelessness, as well as inadequate behavior that can foster the negative circumstances that caused the enhanced anxiety (Brailovskaia & Margraf, 2020b).

In the current study, anxiety served as a significant mediator of the relationship between SMU and burden experience (confirmation of Hypothesis 2). Hence, after the inclusion of anxiety in the mediation model, the direct relationship between SMU and burden was not longer significant. This finding implies that the unfiltered information about COVID-19 provided on SM can foster a negative emotional state of anxiety. Online interaction about such information with friends and strangers can further enhance the state of anxiety, which can contribute to the experience of burden by the COVID-19 situation (Garfin et al., 2020; Ng et al., 2018). Thus, the higher the frequency of SMU as a COVID-19 information source, the higher the individual anxiety level and the higher the risk to experience burden. Individuals who experience high levels of burden typically tend to a maladaptive response such as reduced adherence to governmental measures (Brailovskaia & Margraf, 2020b). This can negatively impact the COVID-19 situation because adherence to governmental measures is vital in the fight against the spread of the pandemic.

After the easing of the governmental measures in May 2020, the daily COVID-19 case numbers remained relatively low and stable in Spain. However, since August 2020, they steady increased. With > 498,900 confirmed cases, Spain was placed 9th in the worldwide COVID-19 ranking on September 04, 2020 (Hasell et al., 2020). After the increase in case numbers starting in August 2020, some restrictions and bans became effective again. Wearing face masks in public transport and in public places is currently compulsory, smoking in public is forbidden if safe distancing of 2 m cannot be maintained, nightclubs and nighttime bars are closed (Ministerio de Sanidad, 2020b). Moreover, some cities and villages are adopting stricter measures (e.g., prohibitions of leaving one’s home city or village, or social gatherings of more than ten people).

Considering the anew increase of daily COVID-19 cases in Spain and in other countries, it is important to identify and to reduce potential risk factors that can foster the experience of burden by the current situation. The present results reveal that anxiety and the use of SM as a source of COVID-19 information might be such risk factors. Therefore, it seems urgent to communicate the need for a stronger regulation of information provided on SM about COVID-19 both by providers and individual users who actively share online content (Pennycook et al., 2020). Before sharing and liking information on Facebook or Twitter, the user should ensure its accuracy – for example by checking whether the same information is provided on official governmental sites. The call for public attention to this issue by government and authorities can contribute to more caution with SM and, therefore, a reduction of the negative impact on the experienced burden. Moreover, SM may be used by the government to share verified information, to reduce anxiety and to stress the need to adhere to rules.

Following limitations of the present study are to be considered. First, its cross-sectional design does not allow conclusions on causality. The replication of the present findings in a longitudinal study is desirable. Second, the high female proportion of the relatively small investigated sample limits the generalizability of the present findings to the general Spanish population. Therefore, future studies are recommended to replicate the current findings in a larger sample that is more social-demographically representative for the population in Spain. Third, the current results are a snapshot of the COVID-19 situation in Spain in the beginning of May 2020. Their replication in other countries and at further time-points is desirable. Fourth, the relatively low internal consistency of the burden measure limits the findings’ reliability. Fifth, only the frequency of media use was assessed. Future studies are suggested to measure the individual evaluation of the information provided on the different forms of media (e.g., as supportive, confusing, credible) to better understand the mechanisms that underly the relationship between media use and burden caused by COVID-19.

In conclusion, the present study shows that SM are commonly used as the COVID-19 information source in Spain. This can foster the experience of burden by the current extraordinary situation. Anxiety that is enhanced by misinformation and fake news often provided on SM mediates the relationship between SMU and experienced burden. Considering that anxiety and high levels of burden can reduce the adherence to governmental measures and, therefore, disrupt the fight against the pandemic, a tighter control of uploading and sharing online content on SM by both providers and individual users is necessary to reduce the spread of COVID-19. This is of specific importance considering the anew increase of daily COVID-19 cases.
